# Short-term mortality risks among patients with non-metastatic bladder cancer

**DOI:** 10.1186/s12885-020-07655-x

**Published:** 2020-11-25

**Authors:** Menghe Zhai, Chenye Tang, Ming Li, Xin Chen, Yigang Jin, Xiangjun Ying, Zhiling Tang, Xiao Wang, Yuntao Wu, Chun Sun, Kean Chen, Xiao Guo

**Affiliations:** Department of Urology, The Second Affiliated Hospital of Jiaxing University, Jiaxing, Zhejiang Province People’s Republic of China

**Keywords:** Bladder cancer, Causes of death, Survivorship, Short-term survival, SEER

## Abstract

**Background:**

Population-based analysis for the short-term non-bladder cancer related mortality among patients with non-metastatic bladder cancer is currently lacking. The objective of the current study was to assess and quantify cause of death after bladder cancer diagnosis.

**Methods:**

The custom Surveillance, Epidemiology, and End Results (SEER) dataset for standardized mortality ratios (SMRs) was utilized to identify 24,074 patients who were diagnosed with nonmetastatic (M0) bladder cancer from 2014 to 2015. SMRs for causes of death were calculated. Risk factors for bladder cancer-specific mortality, competing mortality, second-cancer mortality, and noncancer mortality were determined using either multivariable Cox or competing risk regression models.

**Results:**

Among all the 4179 (17.4%) deaths occurred during the follow-up period, almost half of them (44.2%) were attributed to non-bladder cancer cause, including second non-bladder cancer (10%) and other non-cancer causes (34.2%). The most common noncancer causes of death were heart diseases followed by chronic obstructive pulmonary disease. Patients had a higher risk of death from second malignancies (SMR, 1.59; 95% CI, 1.47–1.74) compared with death from first malignancies in the US general population, and also had higher risks of death from heart diseases (SMR, 1.29; 95% CI, 1.18–1.40) and chronic obstructive pulmonary disease (SMR, 1.52; 95% CI, 1.29–1.79) compared with the US general population. Additionally, some risk factors for competing second malignancies or noncancer mortality were determined, such as age, gender, marital status and treatment modalities.

**Conclusions:**

Death from non-bladder cancer cause contributed to almost half of all deaths in bladder cancer survivors during the short-term follow-up period. These findings can inform medical management and assist clinicians in counseling those survivors regarding their short-term health risks.

**Supplementary Information:**

The online version contains supplementary material available at 10.1186/s12885-020-07655-x.

## Background

Bladder cancer is the sixth most common cancer and the second commonly diagnosed genitourinary malignancy [1], accounting for approximately 4.6% of all newly diagnosed cancer and for approximately 2.8% of cancer directed deaths in the United States. According to the latest cancer statistic report, an estimated 81,190 new cases of bladder carcinoma were expected to be diagnosed in 2018 in the United States nationally, with 17,240 deaths caused by this disease [2]. Increasing age is considered as the main risk factor for bladder cancer, and most of the case occur in people aged over 60 [3]. In addition, smoking and exposure to some industrial chemicals also increase the risk of bladder cancer [4, 5].

On average, approximately 70% of bladder cancers are classified as localized disease or non-muscle-invasive bladder cancer (NMIBC) at diagnosis, whereas the remaining ~ 30% are classified as muscle-invasive bladder cancer (MIBC) [6]. Hence, due to the early diagnoses and the development of treatment modalities, bladder cancer survival rates improved significantly over time [1]. A previously published large population-based analysis reported that the 5-year cancer-specific survival rates was 92 and 50% for localized and regional disease, respectively [7]. Moreover, about 700,000 patients are estimated to be living with the disease in the USA [8]. Considering the elder age and improved lifetime expectance of bladder cancer survivor, more attention should be paid to identify the cancer patients at high risk of dying from non-bladder cancer cause, as well as their risk of a particular cause of death.

Prior studies have reported on the non-cancer causes of death among patients with various cancer (e.g. prostate [9], breast [10], and oropharynx [11]). Moreover, non-cancer causes of death in long-term bladder cancer survivors were also investigated in a previously published study [12]. However, insight regarding the short-term mortality (e.g. second cancer mortality, and non-cancer mortality) risk in relation to bladder cancer survivor is still limited. Moderate treatment modalities and intensive medical management should be considered for patients who had high risk of dying from competing causes of either secondary malignancies or noncancer cause. Therefore, the purpose of the current work is to study short-term (within 3 years) cause of mortality among survivors of bladder cancer. The results identify patients who are at higher risk to die from second non-bladder cancers, as well as who are at higher risk of non-cancer death (and its specific causes). This information could be utilized for treatment selection, patient counseling, and survivorship monitoring.

## Methods

### Database and case selection

Data was obtained from the custom SEER database [Incidence- SEER 18 Regs excluding AK Custom Data (with additional treatment fields), Nov 2018 Sub (2000–2016) for SMRs], which collected cancer data that covers approximately 28% of the United States population [13]. The SEER*Stat software version 8.3.6.1 (National Cancer Institute, USA) was utilized to access the data from SEER database. Patients with non-metastatic (M0) bladder cancer (Site recode International Classification of Diseases for Oncology-3 (ICD-O-3)/WHO 2008: Urinary bladder) with malignant behavior who were diagnosed between 2014 to 2015 were extracted from the database. Only patients with a single primary tumor or patients whose first primary tumor was bladder cancer were included. Patients whose diagnosis was made at autopsy or based on a death certificate were excluded (*n* = 108). Importantly, to minimize the risk of selection bias, eligible patients who have missing information of variables in SEER program were also included for subsequent regression analysis of survival data. A flow chart of inclusions and exclusions was shown in Figure S1.

### Covariates

The analysis involved multiple variables including demographic characteristics (age at diagnosis, gender, race, marital status and insurance status), disease characteristics (histologic grade, AJCC T and N stage, and SEER based summary stage which categorizes tumors into localized, regional, or distant disease), and treatment characteristics (surgery, radiotherapy and chemotherapy). Our use of the radiotherapy and chemotherapy data in this study was consistent with the data use agreement which we signed to obtain those variables. Specially, the continuous variables, age at diagnosis, were transformed into categorical variables (0–49, 50–64, and > 64). Marital status including divorced, single, widowed, separated and domestic partner were classified into other status. The SEER Cause of Death Recode was utilized to identify the causes of death, which was based on the ICD 8–10 (International Statistical Classification of Diseases and Related Health Problems) and included both cancer and non-cancer causes of death. SEER recodes ICD-8 through ICD-10 codes from death certificates and categorizes deaths from noncancer causes in 26 major groups. These groups have been defined consistently over time and include many of the leading causes of death in the United States, such as *pneumonia and influenza*, *diseases of heart*, and *suicide and self-inflicted injury* [14]. Vital status record recode and cause-specific death classification were utilized to define the main outcomes including all-cause mortality and cancer-specific mortality.

### Statistical analysis

Descriptive statistic was utilized to summarize the absolute number and percentage of deaths among bladder cancer patients within each time period. The standardized mortality ratios (SMRs) were directly estimated by SEER*Stat software. In detail, the SMRs (adjusted by variables including age, sex, and race) for each cause of death after bladder cancer diagnosis were defined by calculating the ratio of observed-to-expected (O/E) mortality, which represents the change in the risk for a specific cause of death after bladder cancer diagnosis compared with the risk of the general US population. The observed mortality represents the observed number of deaths attributed to a specific cause within a specific timeframe, while the expected mortality represents the number of death which are expected to attribute to the same cause in a demographically similar population within the same time frame. Subsequently, a multivariable Cox proportional hazards regression model was used to estimate the hazard ratios (HR) for all-cause mortality. Meanwhile, multivariable Fine and Gray competing-risk regression models were built to estimate subdistribution hazard ratios (SHR) for other endpoints, including bladder cancer-specific mortality, competing-cause mortality, second cancer mortality, and noncancer mortality.

Descriptive statistic, and multivariate Cox proportional hazards model were performed using SPSS 24.0 (IBM Corp). The Fine and Gray competing-risk regression model was conducted by using R software version 3.6.0. A 2-sided *P* value of < 0.05 was considered as statistical significance unless otherwise stated.

## Results

### Baseline characteristics

Based on the inclusion criteria, a total of 24,074 patients diagnosed with non-metastatic bladder cancer were extracted from the SEER database. Most of the patients who were included were elderly with age > 64 (69.1%). Most included patients were white (89.0%), and were diagnosed with in situ bladder cancer (53.8%). Moreover, The majority of patients (94.3%) had undergone cancer-directed surgery, whereas only very few patients (4.2%) received radiotherapy. Of all the included patients, a total of 4179 (17.4%) patients were recorded as dead during the follow-up period, with a mean age at death of 77.93 years. It was noted that 2289 deaths (54.8%) occurred within 1 years, while the rest of deaths (1890, 45.2%) occurred range from 1 to 3 years. The baseline characteristics of all patients who were diagnosed with non-metastatic bladder cancer, as well as the time and number of deaths were listed in Table 1.

**Table 1:** Baseline characteristics of all patients with non-metastatic bladder cancer and patients who died according to the time of death after diagnosis

		Timing of deaths after diagnosis					
		All Deaths		< 1 Year		1–3 Years	
Characteristic	Total No. of Patients	No. of Patients (%)	Mean Age at Death, y	No. of Patients (%)	Mean Age at Death, y	No. of Patients (%)	Mean Age at Death, y
Overall	24,074	4,179 (100)	77.93	2,289 (54.8)	78.49	1,890 (45.2)	77.25
Age at diagnosis, y							
0–49	1,037	59 (100)	45.9	31 (52.5)	45.31	28 (47.5)	46.56
50–64	6,394	649 (100)	60.49	303 (46.7)	60.13	346 (53.3)	60.81
> 64	16,643	3,471 (100)	81.73	1,955 (56.3)	81.86	1,516 (43.7)	81.57
Race							
White	21,432	3,639 (100)	78.22	1,991 (54.7)	78.71	1,648 (45.3)	77.64
Black	1,352	306 (100)	73.35	171 (55.9)	74.95	135 (44.1)	71.33
Asian or Pacific Islander	1,201	206 (100)	79.98	108 (52.4)	80.98	98 (47.6)	78.87
American Indian/Alaska Native	89	28 (100)	74.59	19 (67.9)	73.21	9 (32.1)	77.51
Disease stage							
In situ	12,940	935 (100)	80.25	403 (43.1)	80.32	532 (56.9)	80.20
Localized	8,574	2,051 (100)	79.26	1,154 (56.3)	79.91	897 (43.7)	78.41
Regional	1,814	839 (100)	72.56	496 (59.1)	74.26	343 (40.9)	70.11
Histological grade							
Grade I	1,970	161 (100)	79.8	63 (39.1)	78.89	98 (60.9)	80.39
Grade II	6,232	447 (100)	79.07	214 (47.9)	79.18	233 (52.1)	78.97
Grade III	2,278	615 (100)	77.36	357 (58.0)	77.96	258 (42.0)	76.52
Grade IV	9,598	2,244 (100)	76.85	1,236 (55.1)	77.58	1,008 (44.9)	75.96
Cancer-directed surgery							
Yes	22,699	3,739 (100)	77.58	1,996 (53.4)	78.13	1,743 (46.6)	76.95
Radiotherapy							
Yes	1,000	477 (100)	78.69	259 (54.3)	79.34	218 (45.7)	77.92
Chemotherapy							
Yes	7,186	1,290 (100)	73.18	590 (45.7)	73.62	700 (54.3)	72.81

### Short-term mortality risks after bladder cancer diagnosis

**Fig. 1:**
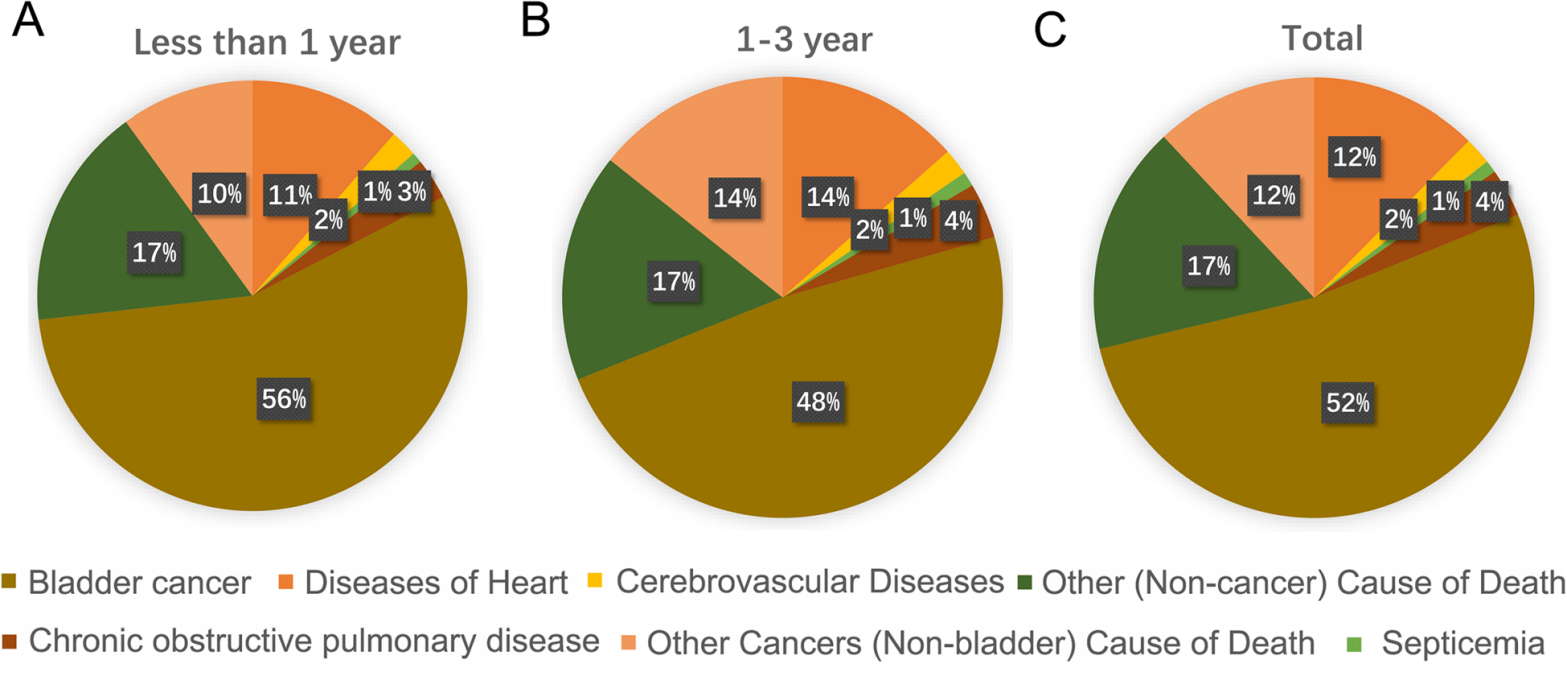
Cause of death in each latency period following bladder cancer diagnosis are illustrated. **a** The proportion of various types of causes of death within 1 year after bladder cancer diagnosis. **b** The proportion of various types of causes of death within 1–3 year after bladder cancer diagnosis. **c** The proportion of various types of causes of death after bladder cancer diagnosis

The proportion of various types of causes of death within 1 year or 1–3 years was displayed in Fig. 1. Among the 2289 patients who died within the first year after bladder cancer diagnosis, a total of 1278 patients (55.8%) died directly from bladder cancer, 229 patients (10.0%) died from other cancers (non-bladder cancer), and 782 (34.2%) patients died from non-cancer causes. It was worth mentioning that patients after bladder cancer diagnosis had a dramatically higher mortality risks (SMRs = 1.50; 95% CI [1.32–1.71]; *P* < 0.05) from non-bladder second cancer within the first year in comparison with the US general population (Table 2). The most common non-cancer cause of death within 1 years was disease of heart (262 death, 11.4%), followed with chronic obstructive pulmonary disease (71 death, 3.1%), both of which had higher number of death than expected death in the US general population, with SMRs of 1.35 (95% CI [1.19–1.52], *P* < 0.05) and 1.52 (95% CI [1.18–1.91], *P* < 0.05), respectively (Table 2). In addition, patients with bladder cancer had a significant higher risk of death from septicemia (SMRs = 1.69; 95% CI [1.00–2.67]; *P* < 0.05), other infectious disease (SMRs = 3.69; 95% CI [2.25–5.69]; *P* < 0.05), pneumonia and influenza (SMRs = 1.67; 95% CI [1.12–2.40]; *P* < 0.05), hypertension without heart disease (SMRs = 1.84; 95% CI [1.05–2.99]; *P* < 0.05), and nephritis, nephrotic syndrome and nephrosis (SMRs = 1.90; 95% CI [1.27–2.72]; *P* < 0.05) within the first year after cancer diagnosis in comparison with the US general population.

**Table 2:** Standardized mortality ratios for each cause of death after bladder cancer diagnosis

	Timing of deaths after diagnosis					
	< 1 Year		1–3 Years		Total	
Cause of death	No. Observed^a^	SMR (95% CI)	No. Observed^a^	SMR (95% CI)	No. Observed^a^	SMR (95% CI)
All causes of death	2289	3.13 (3.00–3.26)^b^	1890	2.41 (2.30–2.52)^b^	4179	2.76 (2.67–2.84)^b^
Bladder cancer-specific death	1278	188.61 (178.41–199.24)^b^	914	124.91 (116.94–133.28)^b^	2192	155.54 (149.09–162.19)^b^
Second-cancer death	229	1.50 (1.32–1.71)^b^	270	1.67 (1.48–1.88)^b^	499	1.59 (1.45–1.74)^b^
Non-cancer causes of death						
Septicemia	18	1.69 (1.00–2.67)^b^	20	1.74 (1.06–2.69)^b^	38	1.72 (1.21–2.36)^b^
Other infectious diseases	20	3.69 (2.25–5.69)^b^	11	1.94 (0.97–3.47)	31	2.79 (1.90–3.97)^b^
Diabetes mellitus	30	1.45 (0.98–2.07)	22	1 (0.62–1.51)	52	1.22 (0.91–1.60)
Alzheimer disease	25	0.76 (0.49–1.12)	27	0.73 (0.48–1.06)	52	0.74 (0.56–0.98)^b^
Diseases of the heart	262	1.35 (1.19–1.52)^b^	255	1.23 (1.08–1.39)^b^	517	1.29 (1.18–1.4)^b^
Hypertension without heart disease	16	1.84 (1.05–2.99)^b^	8	0.85 (0.37–1.68)	24	1.33 (0.85–1.97)
Cerebrovascular diseases	47	1.18 (0.87–1.57)	37	0.86 (0.61–1.19)	84	1.02 (0.81–1.26)
Pneumonia and influenza	29	1.67 (1.12–2.40)^b^	16	0.88 (0.5–1.42)	45	1.26 (0.92–1.69)
Chronic obstructive pulmonary disease	71	1.52 (1.18–1.91)^b^	77	1.53 (1.21–1.92)^b^	148	1.52 (1.29–1.79)^b^
Chronic liver disease and cirrhosis	11	1.67 (0.83–2.99)	9	1.27 (0.58–2.4)	20	1.46 (0.89–2.26)
Nephritis, nephrotic syndrome and nephrosis	29	1.90 (1.27–2.72)^b^	16	0.98 (0.56–1.6)	45	1.43 (1.04–1.91)^b^
Symptoms, signs and ill-defined conditions	6	0.77 (0.28–1.67)	7	0.90 (0.36–1.86)	13	0.84 (0.44–1.43)
Accidents and adverse effects	19	0.86 (0.52–1.35)	19	0.80 (0.48–1.24)	38	0.83 (0.59–1.14)
Suicide and self-inflicted injury	7	1.41 (0.57–2.90)	11	2.07 (1.04–3.71)^b^	18	1.75 (1.04–2.77)^b^
Other cause of death	167	1.34 (1.14–1.56)^b^	147	1.09 (0.92–1.29)	314	1.21 (1.08–1.35)^b^

*Abbreviation*: *SMR* standardized mortality ratio

^a^Values indicate the number of patients with cancer who died from each cause of death

^b^*P* < 0.05

Within 1 to 3 years after bladder cancer, a total of 914 patients (48.4%) died directly from bladder cancer, 270 patients (14.3%) died from other cancers (non-bladder cancer), and 706 (37.4%) patients died from non-cancer causes. In accordance with period of follow-up within 1 year, patients after bladder cancer diagnosis had significant higher risk of death from non-bladder second cancer (SMRs = 1.67; 95% CI [1.48–1.88]; *P* < 0.05), septicemia (SMRs = 1.74; 95% CI [1.06–2.69]; *P* < 0.05), disease of heart (SMRs = 1.23; 95% CI [1.08–1.39]; *P* < 0.05), chronic obstructive pulmonary disease (SMRs = 1.53; 95% CI [1.21–1.92]; *P* < 0.05) compared with the US general population. Conversely, deaths from non-cancer causes including hypertension without heart disease, pneumonia and influenza, Nephritis, nephrotic syndrome and nephrosis, and other infectious disease within 1–3 years after bladder cancer diagnosis were no difference with the general population. Interestingly, our results indicated that patients with bladder cancer had a statistically significant high risk of death from suicide and self-inflicted injury within 1 to 3 years after cancer diagnosis, with SMRs of 2.07 (95% CI [1.04–3.71], *P* < 0.05), but not within 1 year, with SMRs of 1.41 (95% CI [0.57–2.90], *P* > 0.05), when compared with US general population (Table 2).

### Multivariable analysis of risk factors for short-term mortality risk

In order to better understand the possible risk factors associated with bladder cancer-specific mortality, non-bladder cancer mortality or non-cancer mortality, multivariate Cox analysis or competing risk analysis were conducted (Table 3). The multivariate analysis indicated that patients with age within 50–64 (vs age < 50; SHR = 1.47; 95% CI [1.13–1.93]; *P* = 0.005), age > 65 (vs age < 50; SHR = 2.37; 95% CI [1.82–3.08]; *P* < 0.001), Female (vs Male; SHR = 1.26; 95% CI [1.15–1.38]; *P* < 0.001), Black (vs White; SHR = 1.18; 95% CI [1.02–1.38]; *P* = 0.030), American Indian (vs White; SHR = 1.70; 95% CI [1.09–2.63]; *P* = 0.020), T2 (vs T1; SHR = 5.82; 95% CI [5.17–6.55]; *P* < 0.001), T3 (vs T1; SHR = 6.85; 95% CI [5.89–7.97]; *P* < 0.001), T4 (vs T1; SHR = 11.6; 95% CI [9.87–13.7]; *P* < 0.001), N1 (vs N0; SHR = 1.60; 95% CI [1.33–1.92]; *P* < 0.001), N2 or N3 (vs N0; SHR =2.00; 95% CI [1.72–2.33]; *P* < 0.001), Grade III (vs Grade I; SHR = 4.68; 95% CI [3.33–6.57]; *P* < 0.001), Grade IV (vs Grade I; SHR = 3.98; 95% CI [2.86–5.53]; *P* < 0.001), and Radiation (vs No radiation; SHR = 1.30; 95% CI [1.15–1.47]; *P* < 0.001) were significantly associated with poor bladder cancer-specific survival. On the contrary, patients who had factors like insured (vs uninsured; SHR = 0.65; 95% CI [0.49–0.86]; *P* = 0.003), married (vs single; SHR = 0.66; 95% CI [0.58–0.74]; *P* < 0.001), surgery (vs no surgery; SHR = 0.54; 95% CI [0.45–0.65]; *P* < 0.001), and chemotherapy (vs no chemotherapy; SHR = 0.65; 95% CI [0.59–0.71]; *P* < 0.001) were significantly associated with increased bladder CSS. Moreover, the multivariate analysis for overall survival (OS) of those patients showed consistent result with bladder CSS, expect for gender (female vs male). Taken together, these results suggested that patients with older age, female, other race (black or American Indian), uninsured, single, later stage of T and N, poorer grader, receiving radiotherapy, and not receiving surgery or chemotherapy seemed to have bad clinical outcome.

**Table 3:** Comprehensive multivariable-adjusted Hazard Ratios for all-cause mortality, bladder cancer-specific mortality, competing-cause mortality, second-cancer mortality, and Noncancer mortality among patients diagnosed with nonmetastatic bladder cancer from 2014 to 2015

	All-cause mortality		Bladder cancer-specific mortality		Competing-cause mortality		Second cancer mortality		Noncancer mortality	
Characteristic	AHR (95% CI)	*P*	AHR (95% CI)	*P*	AHR (95% CI)	*P*	AHR (95% CI)	*P*	AHR (95% CI)	*P*
Age										
< 50	1.0 (Ref)		1.0 (Ref)		1.0 (Ref)		1.0 (Ref)		1.0 (Ref)	
50–64	1.72 (1.32–2.24)	<.001	1.47 (1.13–1.93)	<.005	2.83 (1.50–5.34)	<.001	10.8 (7.6–14.8)	<.001	2.30 (1.21–4.37)	0.011
65+	3.66 (2.83–4.73)	<.001	2.37 (1.82–3.08)	<.001	8.76 (4.70–16.3)	<.001	33.3 (29.0–38.2)	<.001	7.49 (4.03–13.9)	<.001
Gender										
Male	1.0 (Ref)		1.0 (Ref)		1.0 (Ref)		1.0 (Ref)		1.0 (Ref)	
Female	1.03 (0.96–1.11)	0.412	1.26 (1.15–1.38)	<.001	0.71 (0.64–0.81)	<.001	0.68 (0.49–0.94)	0.021	0.72 (0.63–0.82)	<.001
Race										
White	1.0 (Ref)		1.0 (Ref)		1.0 (Ref)		1.0 (Ref)		1.0 (Ref)	
Black	1.16 (1.03–1.31)	0.013	1.18 (1.02–1.38)	0.030	0.98 (0.79–1.21)	0.850	0.82 (0.45–1.52)	0.53	0.99 (0.79–1.25)	0.970
Asian or Pacific islander	0.95 (0.83–1.09)	0.485	0.88 (0.74–1.06)	0.180	1.05 (0.84–1.30)	0.680	1.12 (0.64–1.96)	0.69	1.04 (0.82–1.32)	0.740
American Indian	1.69 (1.16–2.46)	0.006	1.70 (1.09–2.63)	0.020	1.30 (0.62–2.70)	0.490	2.81 (0.72–10.9)	0.14	1.05 (0.45–2.48)	0.910
Insurance status										
Uninsured	1.0 (Ref)		1.0 (Ref)		1.0 (Ref)		1.0 (Ref)		1.0 (Ref)	
Any Medicaid	0.96 (0.73–1.27)	0.776	0.77 (0.57–1.04)	0.092	1.85 (0.94–3.64)	0.740	1.47 (0.34–6.25)	0.600	2.05 (0.96–4.40)	0.065
Insured	0.72 (0.55–0.94)	0.017	0.65 (0.49–0.86)	0.003	1.30 (0.67–2.52)	0.440	1.49 (0.38–5.93)	0.570	1.40 (0.66–2.97)	0.370
Unknown	0.72 (0.53–0.98)	0.039	1.12 (0.64–1.26)	0.530	0.82 (0.40–1.67)	0.580	0.85 (0.18–3.98)	0.840	0.90 (0.40–2.00)	0.790
Marital status										
Single	1.0 (Ref)		1.0 (Ref)		1.0 (Ref)		1.0 (Ref)		1.0 (Ref)	
Married	0.67 (0.60–0.73)	<.001	0.66 (0.58–0.74)	<.001	0.74 (0.63–0.87)	<.001	1.03 (0.66–1.60)	0.910	0.71 (0.60–0.85)	<.001
Other status	1.10 (0.99–1.21)	0.070	0.99 (0.87–1.12)	0.820	1.27 (1.07–1.50)	0.006	1.59 (0.99–2.53)	0.054	1.26 (1.06–1.51)	0.010
Unknown ^a^	0.69 (0.59–0.80)	<.001	0.65 (0.53–0.80)	<.001	0.83 (0.66–1.05)	0.120	0.91 (0.47–1.74)	0.770	0.84 (0.65–1.07)	0.160
T stage										
Tis, Ta or T1	1.0 (Ref)		1.0 (Ref)		1.0 (Ref)		1.0 (Ref)		1.0 (Ref)	
T2	3.70 (3.40–4.02)	<.001	5.82 (5.17–6.55)	<.001	1.40 (1.20–1.63)	<.001	0.68 (0.43–1.07)	0.096	1.54 (1.31–1.82)	<.001
T3	3.94 (3.49–4.44)	<.001	6.85 (5.89–7.97)	<.001	1.05 (0.80–1.39)	0.730	1.06 (0.94–2.13)	0.860	1.05 (0.77–1.41)	0.780
T4	6.16 (5.42–7.01)	<.001	11.6 (9.87–13.7)	<.001	0.78 (0.54–1.13)	0.190	0.41 (0.14–1.19)	0.100	0.86 (0.58–1.27)	0.440
Unknown	2.02 (1.64–2.48)	<.001	3.25 (2.43–4.35)	<.001	1.15 (0.81–1.63)	0.430	0.74 (0.36–1.49)	0.390	1.23 (0.84–1.82)	0.290
N stage										
N0	1.0 (Ref)		1.0 (Ref)		1.0 (Ref)		1.0 (Ref)		1.0 (Ref)	
N1	1.46 (1.24–1.73)	<.001	1.60 (1.33–1.92)	<.001	0.79 (0.48–1.30)	0.350	0.76 (0.19–3.09)	0.710	0.79 (0.46–1.34)	0.370
N2 or N3	1.93 (1.69–2.22)	<.001	2.00 (1.72–2.33)	<.001	1.11 (0.76–1.62)	0.600	0.83 (0.27–2.54)	0.740	1.14 (0.76–1.70)	0.530
Unknown	1.08 (0.90–1.31)	0.406	1.16 (0.89–1.52)	0.280	1.17 (0.84–1.63)	0.340	2.16 (1.18–3.95)	0.013	1.05 (0.72–1.53)	0.800
Histological grade										
Grade I	1.0 (Ref)		1.0 (Ref)		1.0 (Ref)		1.0 (Ref)		1.0 (Ref)	
Grade II	0.96 (0.80–1.14)	0.610	1.45 (1.02–2.07)	0.380	0.80 (0.65–0.99)	0.037	0.76 (0.45–1.28)	0.300	0.81 (0.65–1.02)	0.070
Grade III	2.05 (1.72–2.45)	<.001	4.68 (3.33–6.57)	<.001	1.07 (0.85–1.35)	0.570	1.42 (0.80–2.52)	0.230	1.04 (0.80–1.34)	0.780
Grade IV	1.83 (1.55–2.15)	<.001	3.98 (2.86–5.53)	<.001	1.09 (0.89–1.32)	0.400	1.00 (0.62–1.63)	0.990	1.12 (0.90–1.38)	0.320
Unknown	1.50 (1.26–1.78)	<.001	2.82 (2.01–3.96)	<.001	1.09 (0.89–1.34)	0.390	1.39 (0.84–2.30)	0.200	1.06 (0.85–1.33)	0.620
Surgery										
No surgery	1.0 (Ref)		1.0 (Ref)		1.0 (Ref)		1.0 (Ref)		1.0 (Ref)	
Surgery	0.50 (0.44–0.57)	<.001	0.54 (0.45–0.65)	<.001	0.57 (0.47–0.70)	<.001	0.51 (0.29–0.88)	0.015	0.58 (0.47–0.72)	<.001
Chemotherapy										
No chemotherapy	1.0 (Ref)		1.0 (Ref)		1.0 (Ref)		1.0 (Ref)		1.0 (Ref)	
Chemotherapy	0.61 (0.57–0.66)	<.001	0.65 (0.59–0.71)	<.001	0.64 (0.56–0.72)	<.001	0.70 (0.50–0.96)	0.028	0.63 (0.55–0.72)	<.001
Radiotherapy										
No radiation	1.0 (Ref)		1.0 (Ref)		1.0 (Ref)		1.0 (Ref)		1.0 (Ref)	
Radiation	1.31 (1.18–1.45)	<.001	1.30 (1.15–1.47)	<.001	1.38 (1.11–1.71)	0.003	1.91 (1.05–3.47)	0.034	1.34 (1.07–1.69)	<.001

*Abbreviations*: *AHR* adjusted hazard ratio, *Ref* reference category

^a^other status including divorced, widowed, separated or domestic partner

As shown in Table 3, increasing age, male, receiving radiotherapy, and other marital status (divorced, widowed, separated or domestic partner) were associated with a greater risk of competing-cause mortality. Patients who had older age or undertaken radiotherapy were more likely to die from both second non-bladder cancer and non-cancer cause (*P* < 0.05), whereas those who had female gender, or received treatment like surgery or chemotherapy were significantly associated with both decreased second non-bladder cancer mortality and non-cancer mortality (*P* < 0.05). Interestingly, our result also suggested that patients who had married status were less likely died from non-cancer mortality than single status (SHR = 0.71; 95% CI [0.60–0.85]; *P* < 0.001). On the contrary, other marital status including divorced, widowed, separated or domestic partner were exhibited as risk factors for elevated non-cancer mortality (SHR = 1.26; 95% CI [1.06–1.51]; *P* = 0.010).

## Discussion

The current study analyzed the short-term mortality in patients diagnosed with bladder cancer. Numerous previously published retrospective studies have analyzed the malignant causes of bladder cancer-specific mortality [15, 16]. Nevertheless, few studies have concentrated on the non-malignant causes of death, especially in the first few years after cancer diagnosed. Although bladder cancer was the most common cause of death during the initial 3 year follow-up, our result showed that non-bladder cancer cause accounted for approximately one-half of early death. In addition, although bladder cancer survivors were at a relatively higher risk to die from second primary malignancies compared with the US general population, non-cancer deaths accounted for the majority of (74.9%) the competing-cause deaths. It was noted that previously published large epidemiological studies generally consider all-cause mortality as the main endpoint for bladder cancer [17, 18]. Hence, our results implied that Hazard Ratios from those epidemiological studies might overestimate effects of the bladder cancer disease on survival of patients with bladder cancer, especially within the initial 3 years after diagnosis during which cancer was often considered as the most common cause of death. Therefore, it is of great significance to address mortality from non-bladder cancer causes when counselling those patients about short-term prognosis.

To the best of our knowledge, this is the first population-based study to evaluate the causes of early mortality among patients diagnosed with bladder cancer in USA. Patients who had high risk of dying from competing causes of either secondary malignancies or noncancer cause should not consider intensive treatment modalities or need intensive medical management during and after treatment. Hence, our findings may help clinician to individualize risk profiles for specific early mortality events, thus contributing to more individualized clinical decision making as well as survivorship planning.

We found a relatively higher risk of a subsequent malignancy among patients with bladder cancer than US general population. We suspect that unhealthy lifestyle, depression, genetic abnormalities, as well as treatment complications may be potential contributing factors. The most common non-cancer causes of death for bladder cancer patients was cardiac disease, of which the SMR was significantly higher than US general population. This could be attributed to the long-term intravesical chemotherapy with anthracyclines [19], which had dose-dependent cardiotoxicity and thus resulted in the development of cardiomyopathy and heart failure [20]. Additionally, since a large proportion of bladder cancer cases can be attributed to modifiable lifestyle factors, unhealthy lifestyle and consequent diagnosis of diabetes type II or other metabolic diseases might also play a role in deaths from cardiovascular disease. It was demonstrated that chronic obstructive pulmonary disease was the secondly most common non-cancer death, with a significantly high SMR both within 1 year or 1–3 years after cancer diagnosis. This result concur with the previous finding that noted higher rates of death from nonmalignant pulmonary disease in long-term bladder cancer survivors [12]. This result may be explained due to the long-term tobacco smoking, which is well demonstrated as the main risk factor for bladder cancer [16, 21]. Moreover, a significant high risk of death from septicemia and other infectious disease within the first year after cancer diagnosis was also showed in this cohort, largely because of the chemotherapy-induced neutropenia which can lead to the development of sepsis. In addition, our result also indicated that the SMR of suicide increases rapidly within 1–3 years after bladder cancer diagnosis. This result was consistent with previously published studies concentrating on investigating suicide among cancer patients, which noted higher suicide rates in bladder cancer survivors [22, 23]. This could be attributed to the high incidence of depression induced by poor quality of life among these patients [24]. Screening for mental disorders as well as suicidal ideation is of great importance during and after the course of medical treatment for bladder cancer patients.

Our analysis showed that elderly patients were at a higher risk for all types of cause of death. Patients who were married had lower risk of bladder cancer-specific mortality and other non-cancer mortality in comparison with patients with single status. This may be partly explained by the fact that marriage provides social support, which has been applied to buffer the effects of stress [25]. The only exception was that the second non-bladder cancer mortality of married patients was similar to single patients. This might be explained by the fact that the incidence of cancer would not change with marital status. In addition, The data showed that the female was associated with higher risks of bladder cancer-specific mortality. This is in contrast to previously published SEER-based studies concentrating on the long-term survivors of bladder cancer [12]. Moreover, our data also suggested that radiotherapy could offer a short cancer-specific survival for bladder cancer patients. This uncommon result may be explained due to the fact of the controversial role of adjuvant radiotherapy in patients with bladder cancer. A randomized phase II trial demonstrated a significant improvement in DFS and OS for chemoradiation compared with chemotherapy alone [26]. On the contrary, a recent systematic review concluded that there was no clear benefit of adjuvant radiation after radical surgery in bladder cancer [27]. Considering the limited data recently, further prospective studies are needed to confirm the exact role of adjuvant radiotherapy in bladder cancer patients. In addition, we also found that patients who had undergone radiotherapy were more likely to die from either second non-bladder cancer or non-cancer cause when compared with those who had not, which could be attributed to the side-effect of radiation.

Inevitably, there are several limitations to this study. Firstly, this analysis is limited by the intrinsic weaknesses of retrospective databases wherein selection bias is inherent. Secondly, using the SEER classification of death could introduces the possibility of error, albeit the SEER minimized error in death attribution by means of using additional patient data, such as primary site and known comorbidities. Additionally, previous studies also have proved the high degree of accuracy of this variable compared to the use of death certificates [28]. Thirdly, the inferences surrounding treatment effects in this study might be biased because SEER program limited clinical data (e.g. comorbidities) that would allow us to control for selection bias. Further relevant prospective studies are warranted. Given that our study conducted a focused analysis of short-term mortality after cancer diagnosis, further studies would be needed to determine how those factors change over time.

## Conclusion

In summary, death from non-bladder cancer cause contributed to almost half of all deaths in bladder cancer survivors within < 3 years after diagnosis. Second malignancies and cardiac disease were the most common causes of non-bladder cancer deaths during this follow-up periods. Some risk factors for competing second-cancer and noncancer mortality were also identified. These findings can provide important insight into the medical management for non-metastatic bladder cancer patients and assist clinicians in counseling those survivors regarding their short-term health risks.

## Supplementary Information

**Additional file 1: Figure S1.** The flowchart of case selection.

## Data Availability

The datasets generated and/or analysed during the current study are available in the Surveillance, Epidemiology, and End Results Program repository, https://seer.cancer.gov/data/.

## Abbreviations

SEER: Surveillance, Epidemiology, and End Results
SMRs: Standardized mortality ratios
NMIBC: Non-muscle-invasive bladder cancer
MIBC: Muscle-invasive bladder cancer
HR: Hazard ratios
SHR: Subdistribution hazard ratios
CSM: Cancer-specific mortality
