# Supplementary figures and images for: Short-term mortality risks among patients with non-metastatic bladder cancer
Source: BMC Cancer. 2020 Nov 25;20:1148. doi: 10.1186/s12885-020-07655-x (PMC7691110; doi:10.1186/s12885-020-07655-x)

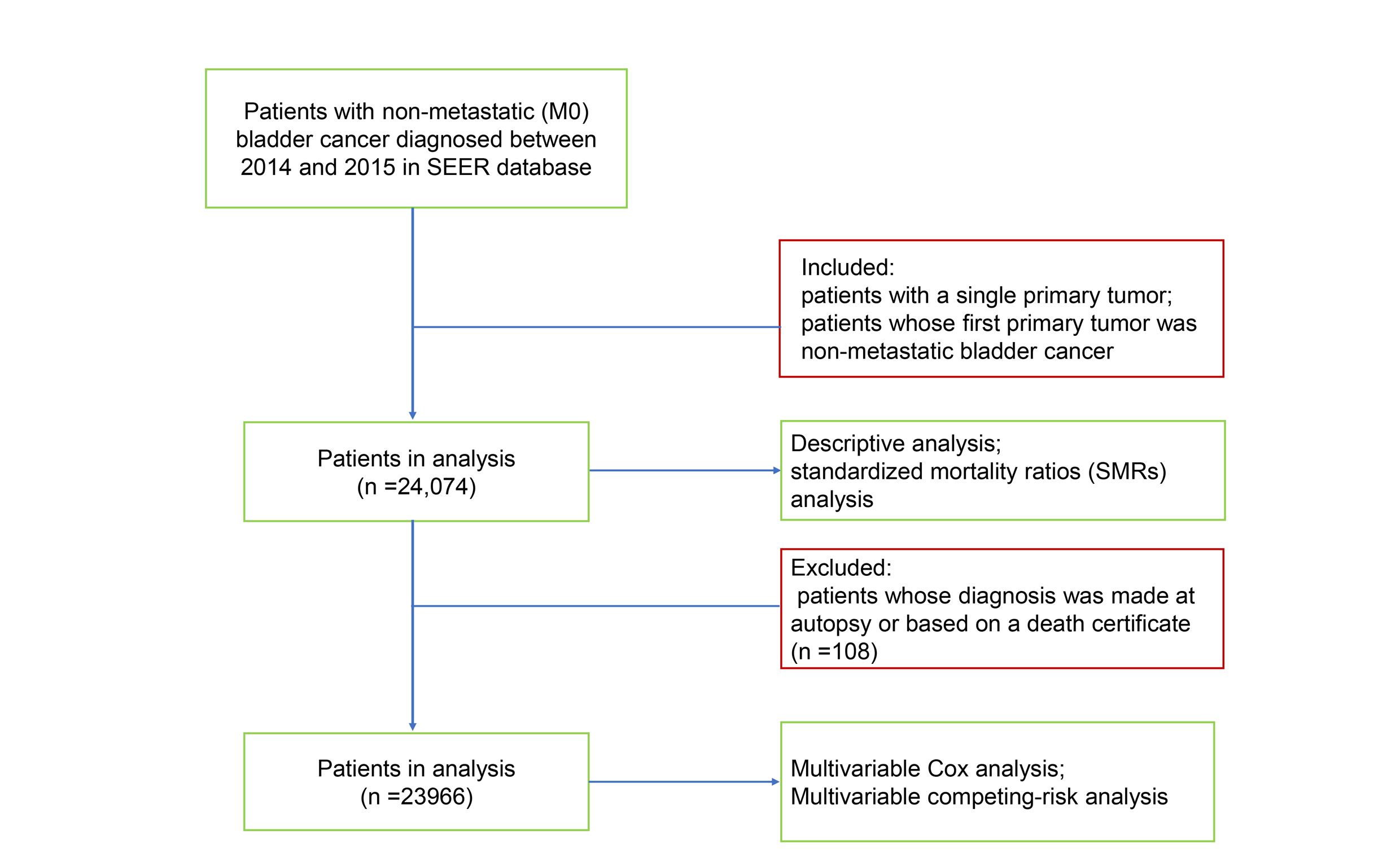

Supplement: Supplementary file 1 — Additional file 1: Figure S1. The flowchart of case selection. [file 12885_2020_7655_MOESM1_ESM.tif]
